# New York Ophthalmic Hospital

**Published:** 1862-06

**Authors:** 


NEW YORK OPHTHALMIC HOSPITAL.—Was removed May 1st, from 63 Third Avenue to 387 Fourth Avenue, corner Twenty-eight street, formerly the country seat of Peter Cooper, Esq., now in a central part of the city, in the immediate vicinity of the medical colleges and near the terminus of the northern and eastern railways.

The old mansion with its spacious halls, rooms and verandas, is admirably adapted for a hospital. It has been recently repaired and is now ready for the reception of patients. Advice and medicine is given to the poor gratuitously, from all parts of the state; those who board in the hospital, pay $3.50 per week.

The attending surgeons are Dr. Mark Stephenson, Dr. J. P. Garrish, and Marcus P. Stephenson; Con. Surgeon, V. Mott, M. D.; Peter Cooper, Esq., President of the Institution.

Communications addressed to either of the above will be attended to promptly. The average attendance of patients is ten hundred per annum, since May, 1862,

